# Zebrafish Renal Pathology: Emerging Models of Acute Kidney Injury

**DOI:** 10.1007/s40139-015-0082-2

**Published:** 2015-04-08

**Authors:** Robert A. McKee, Rebecca A. Wingert

**Affiliations:** Department of Biological Sciences, Center for Zebrafish Research, Center for Stem Cells and Regenerative Medicine, University of Notre Dame, Notre Dame, IN 46556 USA

**Keywords:** Zebrafish, Nephron, Acute kidney injury, Regeneration, Renal progenitor, Renal stem cell

## Abstract

The renal system is vital to maintain homeostasis in the body, where the kidneys contain nephron functional units that remove metabolic waste from the bloodstream, regulate fluids, and balance electrolytes. Severe organ damage from toxins or ischemia that occurs abruptly can cause acute kidney injury (AKI) in which there is a rapid, life-threatening loss of these activities. Humans have a limited but poorly understood ability to regenerate damaged nephrons after AKI. However, researchers studying AKI in vertebrate animal models such as mammals, and more recently the zebrafish, have documented robust regeneration within the nephron blood filter and tubule following injury. Further, zebrafish kidneys contain progenitors that create new nephrons after AKI. Here, we review investigations in zebrafish which have established a series of exciting renal pathology paradigms that complement existing AKI models and can be implemented to discover insights into kidney regeneration and the roles of stem cells.

## Introduction

**Fig. 1:**
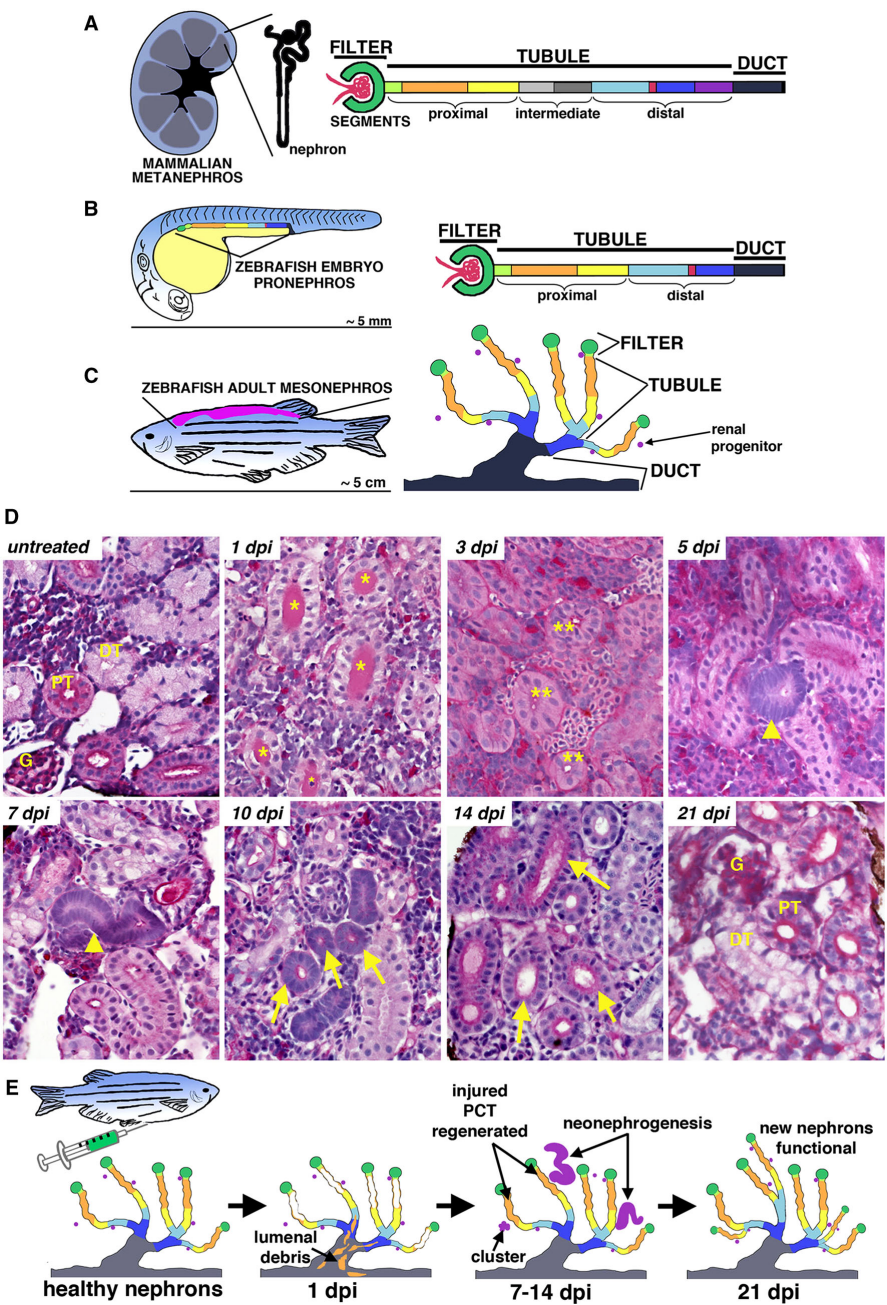
Nephron composition is conserved across vertebrates, and renal regeneration in zebrafish involves two distinct pathological responses. Vertebrate nephron segmental anatomy comparison between a generic mammalian kidney (**a**), zebrafish embryo (**b**), and zebrafish adult (**c**) follows the conserved pattern of blood filter, tubule and duct, where the tubule comprised multiple segments that typically include proximal and distal regions and possibly intermediate segments. **d** Histology time course of kidney regeneration in the adult zebrafish using periodic acid–Schiff staining. Proximal and distal tubule segments (PT, DT) can be distinguished in healthy nephrons based on the deep magenta staining of the PT brush border. AKI was induced by gentamicin injection, and at 1-day post-injury (dpi), DT is filled with casts (*) that have been cleared by 3 dpi (**). From 5 to 14 dpi, basophilic bodies (*arrowheads*) correspond to new nephrons that elongate (*arrows*) to form new nephrons. At 21 dpi, AKI pathology is completely ameliorated, and the tissue is indistinguishable from the uninjured state. **e** Schematic representation of AKI and regenerative events in the zebrafish kidney. [Schematics and images adapted from the following Open Access, Creative Commons Attribution License publications: 23•, 65••]

The kidneys perform a suite of physiological roles that are critical for organismal homeostasis. These tasks include the excretion of metabolites, which are filtered from the circulation to produce urine, as well as the conservation of essential nutrients, osmoregulation, and acid/base balance. The nephron functional units within the kidney are epithelial tubules comprised of a blood filter, specialized proximal and distal segments that modify filtrate composition through solute absorption and secretion, and a duct where water and solute levels are fine-tuned (Fig. 1). An interstitial populace of supporting cells and microvasculature surrounds these nephrons. Kidney tissues can incur damage from a variety of sources that include ischemia and toxins in the bloodstream. Depending on their scope, such injuries can lead to the rapid loss of renal functions over several hours or days, causing a condition termed acute kidney injury (AKI) [1]. AKI is defined clinically as a decreased glomerular filtration rate (GFR) and impaired clearance of waste products, which is diagnosed based on elevated serum creatinine and blood urea nitrogen (BUN) levels, and can be associated with reduced or intermittent urine production [2]. AKI is known to affect primarily the proximal epithelial segments within the nephron tubule, though damage to the glomerular filter, interstitial, and vascular compartments can also initiate an AKI episode [2]. Proximal tubule cells are especially sensitive to reductions in blood supply due to their reliance on aerobic respiration [3]. Further, as proximal tubule cells perform the bulk of solute reabsorption, they preferentially acquire toxins present in the filtrate, leading to regional apoptosis [2, 4]. When nephron cells are destroyed, the resulting cellular debris and protein casts fill the lumen, leading to downstream tubular obstruction and the interruption of fluid flow [2]. Inflammation due to both innate and adaptive immune responses to injury can lead to additional direct insults and destruction of renal tissues [5]. Over time, these parameters can improve in AKI survivors, which historically supported the notion that AKI is a ‘reversible’ condition, at least partially due to regenerative processes in the kidney [5, 6]. However, AKI can lead directly to end-stage renal disease (ESRD), necessitating replacement therapy with dialysis, or an organ transplant to sustain life [7]. Despite the recovery of some patients, AKI has a high morbidity worldwide [7], and AKI-associated mortality in the critically ill patient population ranges between 50 and 80 % [8]. Further, there has been an increasing appreciation that the long-term consequences of AKI include fibrosis and an elevated risk for chronic kidney disease (CKD), in which patients have permanent loss of renal functions [9–11]. Reciprocally, there is a significant risk of AKI in patients with CKD [9–11]. These associations emphasize the significance of understanding the mechanisms of AKI, as well as the need to identify biomarkers and develop therapeutics for this diverse clinical syndrome.

Despite many advances in knowledge about the pathophysiology of AKI in recent years, there remain major unresolved questions about the molecular mechanisms at play during these conditions and how to enhance the native regenerative response of human kidney tissues [12, 13•, 14]. To date, research using animal models has provided many valuable pathological insights into the sequence of events associated with AKI [15]. Mammalian experimental systems using rodents such as the mouse and rat have been shown to mimic various clinical features of human AKI but nevertheless involve some limitations [15]. In recent years, the translational research value of the zebrafish, *Danio rerio*, has been increasingly appreciated based on their high genetic conservation with humans and the development of sophisticated tools for advanced molecular studies [16, 17]. Kidney research using the zebrafish is relevant to the understanding of many aspects of vertebrate renal development and disease based on the formative discovery that fundamental elements of nephron structure and function are conserved (Fig. 1) [18••, 19•, 20•]. During their life, vertebrates form and degrade a series of up to three distinct kidneys forms with differing numbers and arrangements of nephrons, termed the pronephros, mesonephros, and metanephros [21•]. The zebrafish embryo has a simple pronephros composed of two nephrons, while a more complex mesonephros with several hundred nephrons develops during juvenile metamorphosis and becomes the permanent adult organ (Fig. 1) [22•]. Zebrafish nephrons consist of a glomerular filter followed by two proximal and distal tubule segments that drain into common ducts (Fig. 1) [23•]. Further, these cell types show conserved molecular features with their counterparts in the mammalian metanephros, which is the permanent kidney form affected by AKI in humans [23•]. Several zebrafish models of nephron-based AKI have been devised in recent years [23•]. Due to the conserved features of the zebrafish renal organ, these models offer a number of advantages for complementary, novel research on such conditions. For example, studies in the zebrafish embryonic kidney have shown that the nephron tubule can undergo repair through replacement of the epithelium, while studies in the adult kidney have observed a potential for zebrafish to undergo de novo nephrogenesis in response to AKI. In this review, we summarize these and other findings that have established important new avenues for AKI research.

## Studying AKI Pathology in Embryonic Zebrafish

The zebrafish embryo offers several unique opportunities to study the molecular and cellular biology of AKI events, as well as undertake AKI-related drug discovery. Zebrafish embryos undergo rapid, external development and are optically transparent [24], which facilitates their use for organogenesis and disease modeling research. As previously noted, zebrafish nephrons display a conserved segmental composition with other vertebrates [25]. The zebrafish embryo forms an anatomically simple kidney that has two nephrons, which share a common blood filter and a single exit portal for urine at the cloaca [26••]. This simplicity enables experimental access to the renal tissues, which allows for the ablation of specific compartments of the nephron as well as for the ability to observe in vivo changes in real time [25]. Renal organogenesis commences early, with the embryonic nephrons emerging during the first day of development when mesenchymal renal progenitors undergo a cell state transition to form an epithelial tubule [27••, 28]. Tubulogenesis occurs concomitantly with renal progenitor pattern formation, such that the nephron tubule is regionally segmented when it emerges at 24 h post-fertilization (hpf) [29–38]. One distinction from the mammalian pronephros is that the zebrafish pronephric nephrons actively function in the developing embryo, with the onset of blood filtration occurring at approximately 48 hpf [26••], though the filtration sieve undergoes further refinement over 72–96 hpf to become more selective [39]. Taken together, the structure and rapid formation of the zebrafish pronephros enables studies of renal physiology and AKI in a whole organism context [40–43]. As discussed in the following subsections, a number of approaches have been used to induce an AKI phenotype in embryonic zebrafish, ranging from toxins to cell ablation.

### Nephrotoxin Models and Evaluation of Therapeutic Agents Using Chemical Genetics

A seminal study by Hentschel et al. established the first AKI paradigms in zebrafish embryos using gentamicin and cisplatin, molecules that were known to be nephrotoxic in mammals, and showed that fish undergo renal failure with features typical of higher organisms [44••]. The use of gentamicin has since become widely used in the field. The researchers demonstrated that an injection of gentamicin into the cardiac venous sinus of 50 or 72 hpf embryos induced edema in a dose-dependent manner, along with glomerular and tubular distension as well as tubular obstruction due to casts formed by the destruction of tubular cells [44••]. Further, they observed increases in lysosomes within the pronephros 48 h after gentamicin injection, which is a hallmark of aminoglycoside toxicity in mammals [44••]. Subsequent studies have further built upon the characterization of edema, using different classes to indicate categories of morphological severity [45•]. In addition to performing these basic morphological characterizations, Hentschel et al. assessed kidney functionality though the injections of either tetramethylrhodamine 10-kDa dextran or fluorescein-labeled inulin [44••]. Under normal conditions, the kidney will uptake dextran and clear inulin from the body; however, the researchers demonstrated that renal clearance was impaired in fish treated with gentamicin and that there was a reduction in the ability of tubule cells to uptake dextran [44••]. These authors also introduced the idea of simultaneously treating fish with a molecule that could provide a beneficial effect to damage recovery and found that a 4:1 ratio of taurine to gentamicin in injections improved the ability of fish to clear the labeled dextran from their system [44••]. Despite this slight amelioration, treatment with gentamicin or cisplatin ultimately leads to systemic renal failure that is fatal for the embryo [44••].

Subsequent research utilizing chemical genetics led to the discovery that histone deacetylase inhibitors (HDACi) were capable of ameliorating gentamicin-induced AKI in the zebrafish embryo and ischemia–reperfusion (IR)-induced AKI in the mouse [46]. HDACi were initially identified in a small molecule screen for compounds capable of expanding the renal progenitor field in the zebrafish embryo [47••]. The researchers found that embryos treated with the HDACi 4-(phenylthio)butanoic acid (PTBA) or trichostatin A had an expansion of renal progenitors that expressed the genes *lhx1a*, *pax2a*, and *pax8* [47••]. Interestingly, the effect exhibited by these HDACi was similar to previously published work in *Xenopus* with retinoic acid (RA), showing that RA treatment expanded the kidney field [48]. In subsequent studies, treatment with a modified form of PTBA, m4PTB, was demonstrated to attenuate the detrimental effect of gentamicin exposure to the nephron [49••]. Following gentamicin exposure in zebrafish embryos, treatment with m4PTB led to an increased survival rate [49••]. Further, the cell proliferation of tubular cells doubled, while there was not a noticeable effect of m4PTB on levels of apoptosis, suggesting that increased survival rates were due to increased proliferation of renal cells [49••]. Interestingly, m4PTB treatment was also sufficient to enhance AKI recovery in the adult mouse [49••]. Animals that received the chemical displayed less postischemic fibrosis and showed an improved ability to clear creatinine [49••]. The researchers also observed that m4PTB treatment decreased the number of renal tubular epithelial cells in the G2/M phase [49••]. This led them to suggest that the reduction in cell cycle arrest at this stage may account for the observed reduction in fibrosis, a hypothesis that certainly warrants further investigation. Overall, these findings demonstrate the potential for translational drug discovery studies using the zebrafish kidney as a research model.

### Mechanical Obstruction of the Pronephros

Several studies have examined the effects associated with mechanical obstruction of the pronephros tubules in the zebrafish embryo [50•, 51]. Obstruction can be performed using fine tweezers to physically compress, and thus damage, a section of the pronephros tubules or their terminus at the cloaca [50•]. This type of occlusion injury results in the immediate loss of fluid flow within the pronephros and the rapid formation of cystic distension of the tubule within approximately 30 min [50•]. Interestingly, further work revealed that this mechanical obstruction was found to be associated with increased cilia beat rate and dramatic gene expression changes [51]. Namely, researchers discovered that *foxj1a*, which encodes a transcription factor that regulates ciliogenic genes, was upregulated after obstruction in nephron tubule regions where distension had occurred [51]. These findings provided new insights on the response of renal epithelial cells to damage that impacted tubular flow and corroborate increasing evidence that cilia-based mechanosensory signaling is a central component of nephron homeostasis [51].

### Using Localized Laser Ablation to Trigger Focal AKI

While the above studies show the utility of nephrotoxins or mechanical injury to induce various types of AKI in the zebrafish embryo, the use of these techniques is irrevocably associated with lethality several days later in development—presumably because the AKI is too catastrophic. Alternative injury methods utilizing laser ablation have been established and have demonstrated that epithelial regeneration can occur robustly in the zebrafish pronephros when tubular damage is localized [52••, 53, 54]. As previously discussed, due to their optical transparency, it is possible to inject zebrafish embryos with fluorescently labeled dextran molecules that can be monitored as it circulates and becomes absorbed by proximal tubule cells through endocytosis [26, 40]. This technique to fluorescently mark functional proximal tubule cells has been used in our laboratory, in conjunction with a pulsed micropoint laser system, to perform targeted epithelial cell ablation in one nephron [52••]. As the pronephros comprised two nephrons, we adopted a methodology to ablate cells in just nephron, thereby leaving the contralateral side unperturbed to serve as a control as well as retaining a source of renal function for the embryo [52••]. As shown by whole mount in situ hybridization with the proximal tubule marker *slc20a1a*, application of the laser was demonstrated to result in consistent ablation of the targeted area, and that the length of ablation can be well controlled [52••]. Following proximal tubule laser ablation at 72 hpf, tubule integrity was restored by 168 hpf, and the new cells were capable of dextran uptake [52••]. This injury method revealed for the first time that rapid and robust tubule epithelial regeneration can occur in the zebrafish embryo.

A similar ablation technique has been applied to transgenic lines in which a fluorescent reporter was placed under control of a tissue or segment-specific promoter, circumventing the need for dextran uptake-based labeling [53, 54]. The ablation was performed with localized violet laser irradiation using a confocal microscope to assess the effects of nephron cell destruction in ET33d10 and ET-9 transgenic embryonic zebrafish expressing green fluorescent protein (GFP) in the proximal and distal segments of the tubule, respectively [53]. Characterization of this laser photoablation revealed that tubular cell death and proliferation were occurred by 36 h post-injury [53]. Interestingly, through in vivo imaging, collective cellular migration was found to be triggered before this time point such that surviving epithelia moved to fill in the damaged area [53]. Using a series of long (~90 µm) and short (~20 µm) ablation lengths, the researchers uncovered a correlation between the area of damage and collective movement, such that migration was increased when a longer segment of the tubule was destroyed [53]. Additionally, it was shown that the migrating cells likely remained differentiated based on an absence of vimentin staining, the presence of cilia, and intact cell–cell junctional complexes [53]. This study provided the first evidence that collective cell migration is a mechanism of tubule epithelial regeneration and demonstrated that in vivo video imaging can be used in conjunction with this AKI paradigm to capture cellular dynamics in real time.

Further, this photoablation technique has been recently applied to study nephron epithelial regeneration in zebrafish embryos in which researchers compromised formation of the exocyst complex, which mediates the docking and targeting of vesicles in polarized epithelial cells, and is vital for the trafficking of basolateral membrane proteins [54]. Researchers performed antisense knockdown of *sec10*, which encodes a key exocyst component, followed by photoablation tubule injury. They observed that *sec10* knockdown embryos were more sensitive to ablation-induced AKI [54], leading to the proposition that exocyst activity impacts renal cell recovery. This study provides proof of principle that genetic manipulations can be used in conjunction with laser ablation to assess AKI with high resolution in vivo using the zebrafish.

Taken together, the above-mentioned studies illustrate the utility of embryonic zebrafish as a model in which to stimulate AKI through a variety of techniques that allow for detailed characterization of different aspects of tubule repair at the cellular and molecular level. The fact that zebrafish embryos contain only two nephron tubules allows for simple comparisons to be made between damaged and undamaged nephrons, and their optical transparency allows for observation of real time responses to injury. These attributes provide outstanding opportunities for future studies to elucidate new insights into AKI mechanisms through methods that include gene expression analysis [55], forward genetics [56], and the identification of therapeutics with continued chemical genetics [57]. Emergent methods for microscopy in living zebrafish [58] and quantitative renal physiology metric assessments [59, 60] further emphasize the ability to implement novel AKI studies using zebrafish embryos and larvae. However, while young zebrafish embryos perform rapid regeneration of the epithelial nephron tubule, they lack a response seen in the adult kidney: the formation of new nephrons in response to damage or de novo nephrogenesis. The use of adult zebrafish to study nephrogenesis in response to AKI, as well as to model nephron epithelial regeneration in the adult environment, is discussed in the next part of this review.

## The Regenerative Responses to AKI in the Adult Zebrafish Kidney

The adult zebrafish kidney is a single, flattened organ situated adjacent to the dorsal body wall [61]. As the second kidney forms, this mesonephros contains several hundred nephrons and simultaneously serves as the site of hematopoiesis [22•]. The mesonephros begins to develop at approximately 12–14 days post-fertilization [62••, 63••]. These mesonephric nephrons contain a segmented pattern that is highly similar to the nephrons of the pronephros; however, each epithelial tubule contains its own glomerulus, and the distal segments exhibit a greater degree of branching [64••, 65••]. Importantly, the nephrons within the adult zebrafish kidney share histological characteristics and molecular features with nephrons in the mammalian kidney [64••, 65••]. For example, we recently demonstrated that zebrafish nephron proximal tubules display a brush border that can be visualized based on reactivity with periodic acid–Schiff or methanamine silver staining, similar to the mouse [65••].

### Parallels Between Mesonephros Formation and Nephron Formation in Response to AKI

Zhou et al. performed the first in depth analysis of mesonephric development, classifying distinct regions in the adult kidney through live imaging work with transgenic fish strains [62••]. Using individual and interbred transgenic lines that included Tg(*cdh17:EGFP*), Tg(*wt1b:EGFP*), and Tg(*podocin:mCherry*), they showed that mesonephric development beings around 12 days post-fertilization when new nephrons form in close proximity to the region of the pronephros adjacent to the swim bladder [62••]. This has been similarly demonstrated through a recent catalog of whole mount in situ hybridization studies to characterize segment-specific solute transporter expression domains [63••]. Over the next 3 months, renal development progresses with different regions of the kidney generating different quantities of nephrons [62••]. The authors named these regions based on their location along the anterior–posterior axis, as well as the number of nephrons generated during development, as follows: anterior nephron dense region (ANDR), median nephron sparse region (MNSR), median nephron dense region (MNDR), and posterior nephron sparse region (PNSR) [62••]. Additionally, using transgenic lines, they characterized the developmental progression of individual mesonephric nephrons into five stages ranging from an early aggregate to a mature nephron based on the Tg(*wt1b:EGFP*) reporter, in which *wt1b*+ cells became progressively restricted to the urinary pole of the nephron [62••]. Finally, in their study, Zhou et al. injected Tg(*wt1b:EGFP*) fish with gentamicin to induce AKI and then observed reporter gene expression [62••]. Five days after damage, there was an increase in the number of *wt1b:EGFP*+ expressing cell clusters [62••]. This increase in cluster quantity continued until 14 days post-injection [62••]. Taken together, these findings suggested the *wt1b*+ progenitors fuel mesonephros development as well as nephron formation in response to AKI. This is in keeping with the long-held appreciation that in response to AKI, many fish species have the capacity to generate new nephrons, termed de novo nephrogenesis or neonephrogenesis [66••].

Seminal work in a subsequent study illustrated through elegant transplantation methods that nephron progenitors exist in the adult zebrafish kidney and that these progenitors are able to reconstitute new nephrons in response to AKI [67••, 68••]. In their study, Diep et al. sought to establish how the new nephrons were emerging during recovery from gentamicin-induced AKI [67••]. To this end, they isolated whole kidney marrow cells from either Tg(*cdh17:EGFP*) or Tg(*cdh17:mCherry*) fish, which express the respective fluorescent protein reporter throughout the whole tubule [67••]. These donor cells were then transplanted into recipient fish, which were previously immunocompromised by radiation and damaged with an injection of gentamicin [67••]. It was observed that over time, the number of donor-derived nephrons increased as the fish recovered from AKI, and that the new nephrons exhibited functionality based on their ability to uptake a fluorescent 40 kDa dextran [67••]. In seeking to determine if multiple progenitors could engraft and contribute to a single nephron, the authors injected a 1:1 ratio of cells from the adult kidneys of Tg(*cdh17:EGFP*) and Tg(*cdh17:mCherry*) individuals into gentamicin damaged, immunocompromised recipients [67••]. The resulting nephrons exhibited mosaic expression of EGFP and mCherry expressing cells, indicating that multiple nephron progenitors are able to form the same nephron [67••]. To test the proliferative potential of these progenitors, the authors did a serial transplantation experiment and found that in secondary and tertiary transplanted fish, whole kidney marrow cells were still able to engraft and give rise to fluorescently labeled nephrons [67••].

Next, to identify the putative renal progenitor cell populace, the authors examined *lhx1a* and *wt1b*, the zebrafish orthologs of *Lhx1/Lim1* and *Wt1*, respectively, which are transcription factors expressed in developing mammalian nephron pre-tubular aggregates [67••]. Using Tg(*lhx1a:EGFP*) and Tg(*wt1b:mCherry*) strains, the researchers observed that *lhx1a*+ cells appeared just prior to the formation of mesonephric nephrons [67••]. These *lxh1a*+ cells elongated into mature nephrons with *lhx1a* expression becoming restricted as development proceeded [64••]. Similarly, *wt1b*+ cell aggregates were demonstrated to form nephrons, with restriction of *wt1b* expression over time to the glomerulus [67••]. To test whether a single renal progenitor cell could form a mature nephron, additional transplantation studies were performed using single *lhx1a:EGFP*+ cells or aggregates of *lhx1a:EGFP*+ cells [67••]. While individually transplanted cells failed to engraft, aggregates of *lhx1a:EGFP*+ cells were able to give rise to nephrons [67••]. Interestingly, *lxh1a:EGFP*+ cells that were *wt1b*+ failed to engraft, suggesting that this population may be already committed to a specific lineage [67••]. In sum, these studies identified a renal progenitor population in the adult zebrafish kidney that can be isolated based on *lhx1a* expression and is capable of producing nephrons following AKI.

### Nephron Epithelial Regeneration in the Adult Zebrafish Kidney

Recent descriptive studies by our laboratory have chronicled the pathology of cellular events associated with nephron epithelial regeneration following gentamicin-induced proximal tubule injury in the adult zebrafish kidney, chronicling them with respect to the timing of neonephrogenesis (Fig. 1) [65••]. This analysis was enabled by the adaptation of histological and other molecular labeling methods to distinguish tubule segments in zebrafish tissues, such as through their ultrastructural features and differential binding to lectins [64••, 65••]. For example, we established that the zebrafish proximal tubule can be identified based on the presence of a brush border, which can be visualized by hematoxylin and eosin or periodic acid–Schiff staining, along with reactivity to *Lotus tetragonolobus* lectin (LTL) [65••]. These same aspects are routinely used to distinguish the proximal tubule in mammalian kidneys [69] but simply had not been documented in zebrafish. Using these techniques in our study, we found that proximal tubular epithelial regeneration occurred robustly and rapidly, over just one week following the AKI insult. At 1-day post-injection (dpi) of gentamicin, tubular casts were apparent in distal tubules, and LTL+ tubules contained approximately 30 % of dying cells based on staining for nuclear fragmentation with terminal deoxynucleotidyl transferase dUTP nick end labeling (TUNEL) [65••]. Cell death continued at 3 and 5 dpi, with a peak of approximately 40 % of LTL+ cells showing TUNEL co-labeling at 3 dpi [65••]. Concomitant with this wave of cell death, we discovered a partially overlapping wave of cell proliferation based on PCNA reactivity in LTL+ proximal tubules, which began at 2 dpi, peaked to include approximately 60 % of cells at 5 dpi, and declined at 7 and 10 dpi [65••]. Interestingly, we found that mesenchymal cells in regenerating nephrons expressed the *Pax2* transcription factor [65••]. This recapitulates a well-known feature of the regenerating mammalian nephron following AKI [70] and has been similarly observed in the zebrafish embryo following gentamicin injury [49••]. Interestingly, while cellular restoration occurred in the first week subsequent to AKI, widespread restoration of proximal tubule function took substantially longer, with only a subset of nephrons showing dextran uptake at 7 dpi but most by 21 dpi [65••]. Taken together, these observations provide a fundamental framework of the pathological events of zebrafish adult AKI and will be useful in future mechanistic studies.

## Emerging Models of Cell-Type Specific Inducible Injury in Zebrafish

While traditional AKI studies in zebrafish have utilized gentamicin, inducible models of injury have also been developed and represent an exciting new tool to control damage in a temporal and spatial manner. Researchers have now formulated transgenic lines in which conditional, targeted cell ablation can be achieved by utilizing tissue specific expression of the bacterial Nitroreductase (Ntr) enzyme. The transgenic line is made such that the target tissue expresses Ntr [71–73]. To induce cell death, the transgenic fish are exposed to the prodrug metronidazole, which will be up taken by virtually all tissues of the organism [71–73]. In the presence of Ntr, the metronidazole is processed into a DNA cross-linking agent that causes cell destruction [71–74]. To date, nephrology researchers have used this transgenic technology to selectively ablate podocytes, which are specialized epithelial cells that form the glomerular filter, in order to assess podocyte regeneration [75]. Specifically, a Tg(*podocin:ntr*-*mCherry*) line was engineered in which damage could be induced specifically in the podocytes of the glomerulus [76]. Treatment with metronidazole led to severe edema, as well as apoptosis within the glomeruli, indicating a failure of renal function [76]. Furthermore, metronidazole-treated Tg(*podocin:ntr*-*mCherry*) fish exhibited leakage in which large proteins were capable of entering into the nephron tubule [76]. A similar line, Tg(*podocin:ntr*-*GFP*), was used in an independent study in which fish were treated with metronidazole for either 12 or 72 h [77]. At 12 h post-treatment, glomeruli displayed foot process disruption, as well as chromatin condensation [77]. This was exacerbated in fish treated for 72 h that exhibited a complete loss of foot processes and had significantly reduced functional podocytes; however, stopping treatment with metronidazole for 7 days resulted in the reappearance of GFP, suggesting glomerular repair [77]. These research reports illustrate the power of transgenic systems to manipulate cellular viability and hold significant promise for future AKI modeling. This method can be used to make models of cell injury across all nephron cell types, using identified promoters or taking advantage of the powers of recombineering with BACs, which does not necessitate promoter isolation [78], to further expand the types of AKI which can be modeled.

## Conclusion

Despite a rich classical literature documenting the existence of nephron regeneration across vertebrates, the genetic and molecular mechanisms have remained elusive. The studies discussed here illustrate how models of AKI have been created in embryonic and adult zebrafish models in recent years. Taken together, these reports show that a wide variety of methods exists to induce an AKI-like phenotype in zebrafish including injection of nephrotoxins such as gentamicin and cisplatin, laser ablation, and the use of transgenic lines expressing the bacterial nitroreductase in specific tissues. These methods of damage induction have been used in tandem with chemical screens to identify molecules that can convey a pro-regenerative effect and enhance renal recovery, leading to the discovery that exposure to HDACi compounds can ameliorate kidney injury. The field has recently made significant strides in characterizing nephron anatomy and the architecture of the adult mesonephros. Recent work highlights that zebrafish adult nephrons undergo robust epithelial regeneration and the formation of nephrons de novo, the latter that sets zebrafish apart from mammals. Future studies are likely to employ these damage methods as well as new paradigms such as transgenic lines in which injury can be induced in the tubular cells via the nitroreductase–metronidazole system, to further characterize how individual populations of cells in the nephron respond to damage. For example, zebrafish continue to be used as a valuable organismal setting in which to identify nephrotoxins and evaluate their effects [79–83]. As many regenerative phenomena at least partly involve the recapitulation of developmental pathways [84, 85], a complementary and powerful future approach to those noted here will be to explore the roles of nephrogenesis factors in renal regeneration. Additionally, exploration of how reprogramming might be invoked to elicit desirable cell behaviors in vivo or create in vitro sources of replacement cells [86]. The continued use of zebrafish to investigate AKI presents great promise for systematically unraveling the complexities of this diverse clinical condition, as well as identifying potent regenerative therapeutics to treat these conditions.
